# Non-anticoagulant heparin attenuates histone-mediated platelet–leukocyte aggregation and neutrophil extracellular trap formation in a canine whole blood model

**DOI:** 10.3389/fvets.2026.1778453

**Published:** 2026-04-15

**Authors:** Maria A. Guzmán, Lunden Simpson, Ronald H. L. Li

**Affiliations:** 1 Department of Clinical Sciences, College of Veterinary Medicine, North Carolina State University, Raleigh, NC, United States; 2 Feline Health Center, College of Veterinary Medicine, North Carolina State University, Raleigh, NC, United States

**Keywords:** immunothrombosis, inflammation, NETs, non-anticoagulant heparin, platelet–leukocyte aggregate, platelet–neutrophil aggregate, sepsis

## Abstract

**Introduction:**

Sepsis and systemic inflammatory response syndrome (SIRS) are major contributors to morbidity and mortality in dogs, with limited therapeutic advancements. A key factor in this disease progression is the excessive formation of neutrophil extracellular traps (NETs). This study aimed to evaluate the ability of non-anticoagulant heparin (NACH) and unfractionated heparin (UFH) to modulate NET formation and platelet–leukocyte, platelet–neutrophil interactions in an *in vitro* canine model of septic and aseptic inflammation. We hypothesized that NACH would be non-inferior to UFH in attenuating histone-mediated NET formation and platelet–leukocyte, platelet–neutrophil aggregate formations.

**Methods:**

Eleven healthy staff-owned dogs were enrolled after confirmation of normal physical examinations and complete blood counts. Whole blood was collected and incubated with either calf thymus histone (0.5 mg/mL) or heated-killed *E. coli* O111:B4 (1 × 10^6^) to simulate SIRS and sepsis, respectively. Samples were then treated with increasing concentrations of UFH (0, 0.4, 4 and 40 U/mL) or NACH (0, 1, 5, 10 μg/mL). Platelet–leukocyte and neutrophil–platelet aggregates were identified via flow cytometry using fluorophore-conjugated antibodies against platelets (CD61), leukocytes (CD18), and canine neutrophils. NET formation was assessed by quantifying cell-free DNA and intracellular citrullinated histones H3 (citH3) by flow cytometry.

**Results:**

NACH at 10 μg/mL significantly reduced histone-induced platelet–leukocyte (29.0% ± 22.5) and platelet–neutrophil (22.7% ± 15.1) aggregates compared to vehicle controls (37.7% ± 23.7 and 31.8% ± 20.9; *p* = 0.045). UFH did not significantly reduce histone-induced platelet–leukocyte interactions but showed a dose-dependent reduction in *E. coli*-induced platelet–leukocyte aggregates, with 40 IU/mL being the most effective (UFH 40 U/mL = 13.65%, IQR: 9.0 to 24.05 vs. UFH 0 U/mL = 18.10%, IQR: 12.68 to 36.18, *p* = 0.047). Both NACH and UFH modulated neutrophil citH3 expression in *E. coli* model, but only high-dose NACH was able to module neutrophil extracellular trap formation in the histone mediated model.

**Conclusion:**

NACH effectively reduces histone-induced leukocyte and neutrophil–platelet aggregation, while UFH is more effective against *E. coli*-mediated responses. Both forms of heparin modulate NETs formation, highlighting their distinct stimulus-specific anti-inflammatory effects.

## 1 Introduction

Sepsis and systemic inflammatory response syndrome (SIRS) are considered the most challenging conditions in canine medicine due to their variable disease phenotypes and high morbidity and mortality. Mortality rates in small animal (dogs and cats) veterinary patients range from 20–68% for sepsis (1) and approximately 17% in dogs and 40% in cats with SIRS (2). In veterinary medicine, sepsis is defined as a septic focus resulting in SIRS, which is diagnosed by at least two of the four SIRS criteria (3). Recent advances in our understanding of sepsis pathophysiology have challenged this traditional sepsis definition. In human medicine, sepsis is now defined as a dysregulated host response to infection leading to life-threatening organ dysfunction (4). In contrast, SIRS is defined as a clinical syndrome characterized by overwhelming systemic inflammation, triggered by non-infectious causes (2). Common causes of SIRS in dogs include pancreatitis, polytrauma, burns, heat stroke, neoplasia, immune-mediated disease or major surgery among others (2).

During systemic inflammation, the intricate and complex interactions between innate immunity and the coagulation system trigger immunothrombosis. In homeostasis, immunothrombosis provides an effective first-line defense against pathogens by initiating controlled and localized thrombosis, thereby restricting dissemination, and preventing tissue invasion of microorganisms (5). However, its dysregulation, prevalent during inflammatory states such as sepsis or SIRS can contribute to organ dysfunction and worsening clinical status. In addition to degranulation and phagocytosis, neutrophils play an important role in mediating immunothrombosis by releasing neutrophil extracellular traps (NETs), which are decondensed cell-free DNA (cfDNA) decorated with histones and granular proteins, such as myeloperoxidase and neutrophil elastase. NETs not only ensnare microorganisms to facilitate their killing, but they also promote localized thrombosis by activating coagulation and inhibiting fibrinolysis. Platelets, as the effector cells of hemostasis, also function as innate immune cells by directly and indirectly interacting with pathogens and danger or pathogen-associated molecular patterns (PAMPs/DAMPs) (6–8). Upon activation, platelets interact with neutrophils, to further promote NET formation, and other innate immune cells, causing increased platelet action and activation of coagulation (9). During sepsis or SIRS, dysregulation in immunothrombosis leads to overzealous NET formation, resulting in microcirculatory damage, microvascular thrombosis, and ultimately, tissue injury and multiple organ dysfunction (10, 11). Dampening NET production or dismantling certain NET components such as cell-free DNA, nucleosomes, histones or cytotoxic granular proteins could be potential therapeutic targets to improve the mortality of sepsis and SIRS in dogs (12). Despite these conditions being a major contributor to canine morbidity and mortality, advances in their management have remained stagnant in the past decades. Therefore, there is a critical need to identify novel therapeutic targets so that life-saving therapies can be developed to improve patient outcomes (13, 14).

Heparin is a negatively charged polymer that is commonly used due to its anticoagulant properties in patients with hypercoagulable state or hypercoagulable tendency due to comorbidities. Human *in vitro* and *in vivo* studies demonstrated that unfractionated heparin (UFH) also possesses anti-inflammatory and anti-complement properties, which could be beneficial in critically ill dogs (15, 16). The anti-inflammatory properties of UFH arise from its ability to interact and neutralize key proteins such as cytokines, growth factors, cytotoxic peptides and tissue-degrading enzymes involved in mediating systemic inflammation (17). A previous *in vivo* endotoxin model in rats demonstrated a significant decrease in inflammatory cytokines such as tissue necrosis factor-alpha, interleukin 6, and interleukin 8 after preconditioning with different types of heparins. Additionally, lung tissues collected at different time points in affected rats showed significant modulation in tissue injury, due to decreased shedding of the endothelial glycocalyx (16). This protective mechanism is proposed to be due to heparin’s inhibitory effect on heparinase, which plays a role in endothelial glycocalyx shedding, and thereby, mitigates neutrophil adhesion and activation (18). Additionally, heparin’s anti-inflammatory properties may also be related to nuclear factor-kB signaling pathway downregulation and its ability to bind to histones.

Another proposed protective mechanism of heparin in SIRS and sepsis is its inherent ability to bind to free circulating histones, which are positively charged nucleosome proteins released systemically as a result of cellular necrosis and NET formation. As DAMPs, free histones induce apoptosis, systemic inflammation and immunothrombosis by directly priming platelets to augment thrombin generation (15, 19). Since excessive NET formation has been documented in dogs with sepsis and severe inflammatory diseases like immune-mediated hemolytic anemia (IMHA), the use of heparin to modulate NET production and target their components like histones and HMGB1 presents a compelling avenue for further therapeutic research (20, 21).

Despite the widespread use of UFH, its anticoagulant activity can be considered an undesirable property, particularly in critically ill patients with hemostatic derangements. Bleeding tendencies and coagulation abnormalities have been reported in up to 50 to 70% of human patients with critical illness like sepsis; similar data have not been well documented in veterinary patients despite previous studies indicating both hyper and hypocoagulability (16, 22, 23). Evidence suggests that higher doses of UFH are required to achieve its anti-inflammatory effects compared to standard anticoagulant doses, which may increase bleeding risk (15). As a result, there is considerable interest in both human and veterinary medicine in creating and developing non-anticoagulant heparin (NACH) for clinical use that not only retain its anti-inflammatory properties but also reduce or eliminate its anticoagulant activity. A recent study evaluating the use of desulfated heparin, a derivative of NACH, in dogs with septic peritonitis demonstrated safety with escalating doses; however, no significant effects on circulating cfDNA were observed (13). Despite this recent research, a knowledge gap remains regarding the use of NACH in dogs with sepsis and SIRS. Additionally, further research is needed to optimize NACH dosing and formulations to maximize therapeutic efficacy in dogs (13). Currently, several NACH formulations exist, most of which are desulfated heparin derivatives. While the sulfated structure of heparin confers a negative charge that enables interactions with antithrombin, desulfation can result in variable and reduced effectiveness in histone inhibition (17). Studies have shown that selectively desulfated heparins could safely and effectively retain their histone binding capacity (15, 20, 24).

Since current treatment strategies in dogs with sepsis and SIRS do not target immunothrombosis such as NETs, there remains a critical need to evaluate novel therapeutics to improve patient outcome. Our primary objective, therefore, was to evaluate the effects of various concentrations of a partially desulfated NACH and standard UFH on platelet–leukocyte, platelet–neutrophil interactions, and NET formation in canine whole blood treated with *Escherichia coli* (*E. coli*) or histones. We hypothesized that NACH would be non-inferior to UFH in attenuating histone-mediated NET and platelet–neutrophil aggregate formation, while both heparin derivatives would have variable effects on *E. coli*-mediated NETs formation. The NACH selected for the study is characterized by being partially desulfated, and we anticipated that it should retain its anti-inflammatory properties.

## 2 Materials and methods

### 2.1 Animals

A total of 11 clinically healthy staff-owned dogs were enrolled in this study. All animals underwent a physical examination and a complete blood count (CBC). Dogs were included in the study if they did not have any prior medical history, had normal CBCs, and did not receive any medications 30 days prior to blood collection. This study was approved by the Institutional Animal Care and Use Committee at the College of Veterinary Medicine, North Carolina State University (18338).

### 2.2 Blood collection

After obtaining written informed consent from owners, blood was collected via a direct non-traumatic venipuncture on the saphenous, cephalic or jugular vein. Approximately 6 mL of blood was collected and immediately aliquoted into blood tubes containing 3.2% sodium citrate and gently inverted 4 to 5 times to mix. Blood tubes were then placed on a rocker for 15 min at room temperature. Prior to analysis, blood was physically inspected and excluded for further analysis if there was any presence of blood clots. A CBC in a separate aliquot of citrated whole blood was conducted using an automated hematology analyzer (Micro-Cell II Dymind Hematology Analyzer, MicroVet Diagnostics, Las Vegas, NV), and peripheral blood smear evaluation was performed. All samples were processed within 30 min of collection. Blood cell counts were corrected by a factor of 1:1.

### 2.3 *In vitro* Gram-negative sepsis and inflammation model

**Figure 1:**
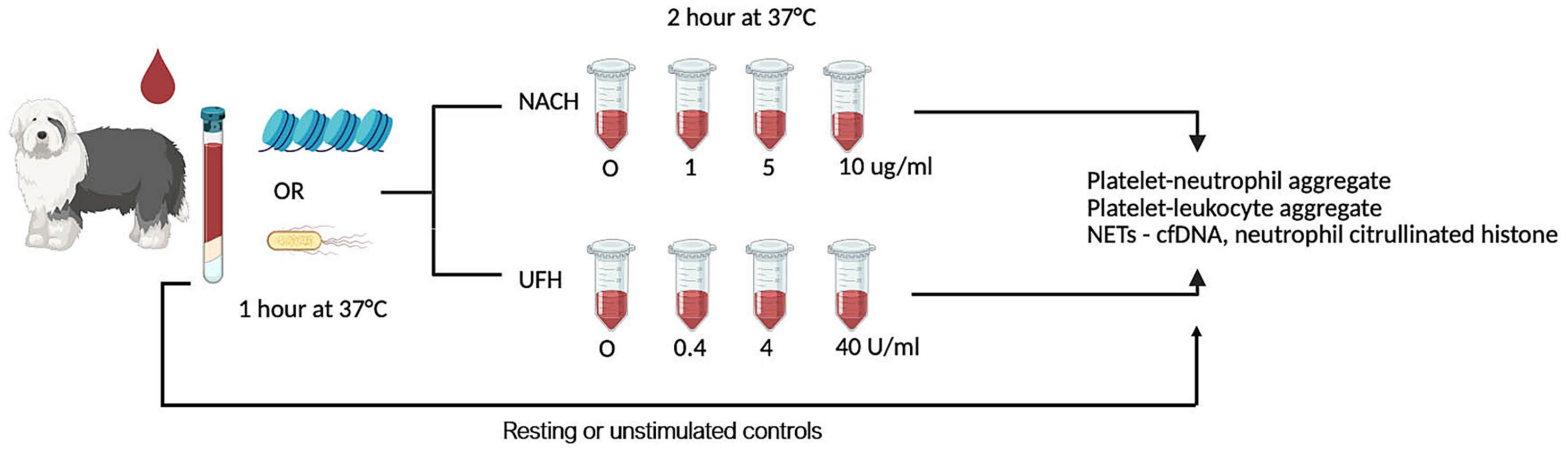
Diagram summarizing study design. Diluted and recalcified citrated whole blood was first treated with calf-thymus histones or *E. coli* for 1 h before the addition of increasing concentrations of non-anticoagulated heparin (NACH) or unfractionated heparin (UFH) for an additional 2 h. Blood samples were then analyzed for platelet–neutrophil aggregates, platelet–leukocyte aggregates, and neutrophil extracellular trap formation via flow cytometry. Diluted whole blood, not treated with histones or *E. coli*, served as resting or unstimulated controls.

Leukocytes (WBCs) were corrected and standardized according to the assays described below. First, to assess platelet-leukocyte or -neutrophil aggregates, WBCs were standardized to 1 × 10^6^ cells/mL using Dulbecco’s phosphate buffered saline (DPBS) with 0.9 mM CaCl_2_ and 5.5 mM dextrose (pH 7.4, 37 °C). To measure neutrophil extracellular trap (NET) formation, WBC was standardized to 2 × 10^6^ cells/mL in the same buffer. Undiluted whole blood was utilized for evaluations of CBCs. Diluted and undiluted whole blood was then treated with the presence or absence of 0.5 mg/mL calf thymus histones (Sigma-Aldrich, Saint Louis, Missouri) or 1 × 10^6^ heat-killed *O111:B4 E. coli* (Invivogen, San Diego, CA) in 100 μL aliquots of whole blood and incubated for 1 h at 37 °C. Thereafter, samples were treated with increasing concentrations of NACH (0, 1, 5, 10 μg/mL) (gift from Rensselaer Polytechnic Institute, Troy, NY, United Staes) or UFH (0, 0.4, 4, 40 U/mL) (Meitheal Pharmaceuticals, Chicago, IL) for an additional 2 h at 37 °C. All dilution series of NAH and UFH were performed using endotoxin-free water (Invivogen, San Diego, CA) to ensure that the same volume of heparins were added to each treatment. Equivalent volumes of endotoxin-free water served as vehicle controls. All experiments were carried out in a sterile manner. Figure 1 depicts the *in vitro* design utilized in this study.

### 2.4 Evaluation of platelet–leukocyte and platelet–neutrophil aggregates

To evaluate platelet–leukocyte (PLA) and platelet–neutrophil aggregates (PNA), immunolabeling of leukocytes, neutrophils and platelets was conducted as previously described (25). In brief, diluted whole blood was labeled with mouse monoclonal antibody to human platelet integrin beta-3 (CD61) conjugated to R-phycoerythrin (1:500, clone:VI-PL2, Invitrogen, Carlsbad, CA), and mouse monoclonal anti-dog antibody to integrin beta-2 (CD18) conjugated to Alexa Fluor 647 (1:40, clone:CA1.4E9, Bio-Rad, Hercules, CA). Neutrophils were labeled with monoclonal anti-dog neutrophil antibody conjugated to fluorescein isothiocyanate (1:200, clone: CAD048A, Washington State Monoclonal Antibody Center, Pullman, WA). Following incubation for 45 min at 37 °C, lysis of erythrocytes and fixation of remaining cells was performed (1X FACS-lyse, BD Biosciences, Milpitas, CA). Samples were stored at 4 °C and protected from light. Flow cytometry analysis occurred within 7 days.

### 2.5 Neutrophil extracellular traps measurement

#### 2.5.1 Neutrophil citrullinated histone expression

After incubation with heparins and vehicle controls, diluted whole blood was first fixed (1X FACS-lyse buffer, 30 min, room temperature), and briefly permeabilized (2% NP-40, ThermoScientific, Rockford, IL). Cells were immediately washed (12,500 × g, 1 min, room temperature) and resuspended with DPBS (no calcium). If no pellets were visible after the first centrifugation step, it was repeated one additional time. Neutrophils were then labeled with rabbit anti-human anti-histone H3 conjugated to allophycocyanin (1:200, clone: ab5103, Abcam, Waltham, MA), known to cross-react with canine citrullinated histone H3 (26), and monoclonal anti-dog neutrophil antibody conjugated to fluorescein isothiocynate (1:200, clone: CAD048A, Washington State Monoclonal Antibody Center, Pullman, WA) and incubated for 45 min at room temperature. Positive control consisted of cells activated with 100 nM phorbal 12-myristate 13-acetate (Millipore Sigma, Burlington, MA) and 200 μM GSK 484 hydrochloride (Abcam, Waltham, MA) treated cells served as negative controls in the presence of heat-killed *E. coli* or calf thymus histones for 1 h at 37 °C. Samples were analyzed within 48 h on the flow cytometer.

#### 2.5.2 Cell-free DNA quantification via spectrophotometry

Following activation and treatment with heparins or vehicle controls, plasma in diluted whole blood was separated by centrifugation at 1,500 RPM (15 min, 4 °C) and stored at −20 °C for future analysis. Plasma cfDNA was analyzed as previously described (26). In short, plasma was thawed at room temperature and then protein was digested with 15 ng proteinase K (Thermo Scientific, Rockford, IL) overnight at 55 °C. RNA was then removed with 0.9 mg RNAse A (37 °C, 5 min) (QIAGEN, Hilden, Germany). Proteins were then precipitated (Puregene protein precipitation solution, Qiagen, Venlo, The Netherlands) and concentrated by centrifugation (16,000 × g, 2 min, at room temperature). The DNA-containing supernatant was extracted after placement on ice and precipitated using 100% isopropanol. After mixing and centrifugation (16,000 × g, 5 min, room temperature), DNA pellet was washed in 70% ethanol and resuspended in 1× Tris-EDTA solution (Fisher BioReagents, Pittsburg, PA). Cell-free DNA concentration was quantified using spectrophotometry in duplicates (Nanodrop 2000c spectrophotometer, Fisher Scientific, Waltham, MA) and the average concentration was calculated. Purity of DNA was determined by 260 to 280 ratio of approximately 1.8.

The magnitude of cfDNA release was assessed using the following equation:


$$\text{cfDNA}\;(\%\text{change})=\frac{\left([\text{cfDNA treatment}]-[\text{cfDNA rest}]\right)}{\left[\text{cfDNA rest}\right]}\times 100$$

rest = untreated cells.

### 2.6 Flow cytometry analysis

**Figure 2:**
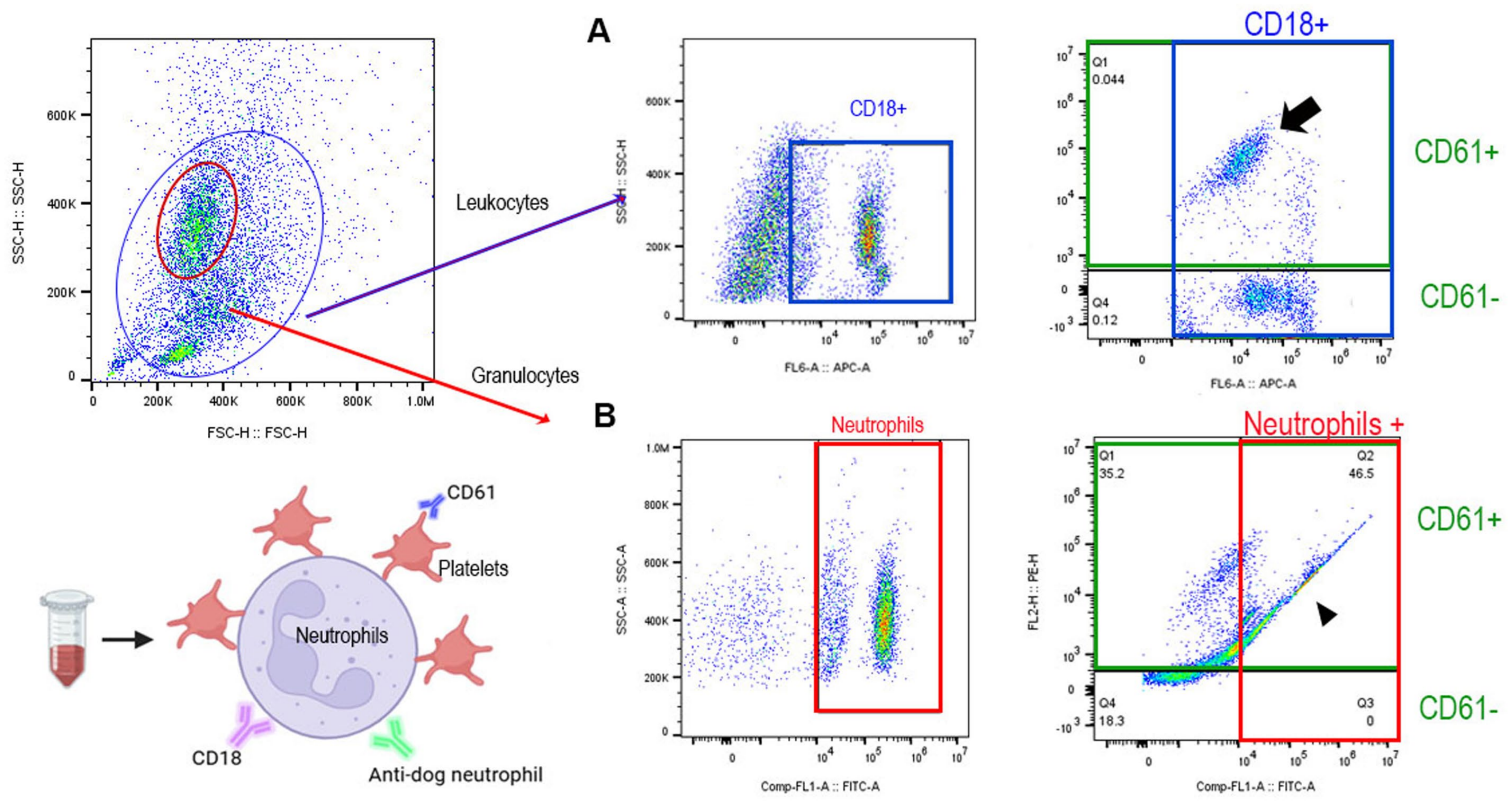
Representative scattered plots of diluted whole blood on flow cytometry. Gating of granulocytes (red) and leukocytes (teal) was established according to side and forward scatter properties. **(A)** Neutrophils were further gated and identified by immunodetection of anti-dog neutrophil marker (red box). **(B)** To identify platelet–neutrophil aggregates (arrowhead), CD61 positive neutrophils (green box) were quantified as % of total neutrophils. **(C)** Leukocytes were characterized by immunodetection of CD18^+^ cells within the leukocyte gate (blue gate). Platelet–leukocyte aggregates (arrow) were identified as CD61^+^ and CD18^+^ cells.

**Figure 3:**
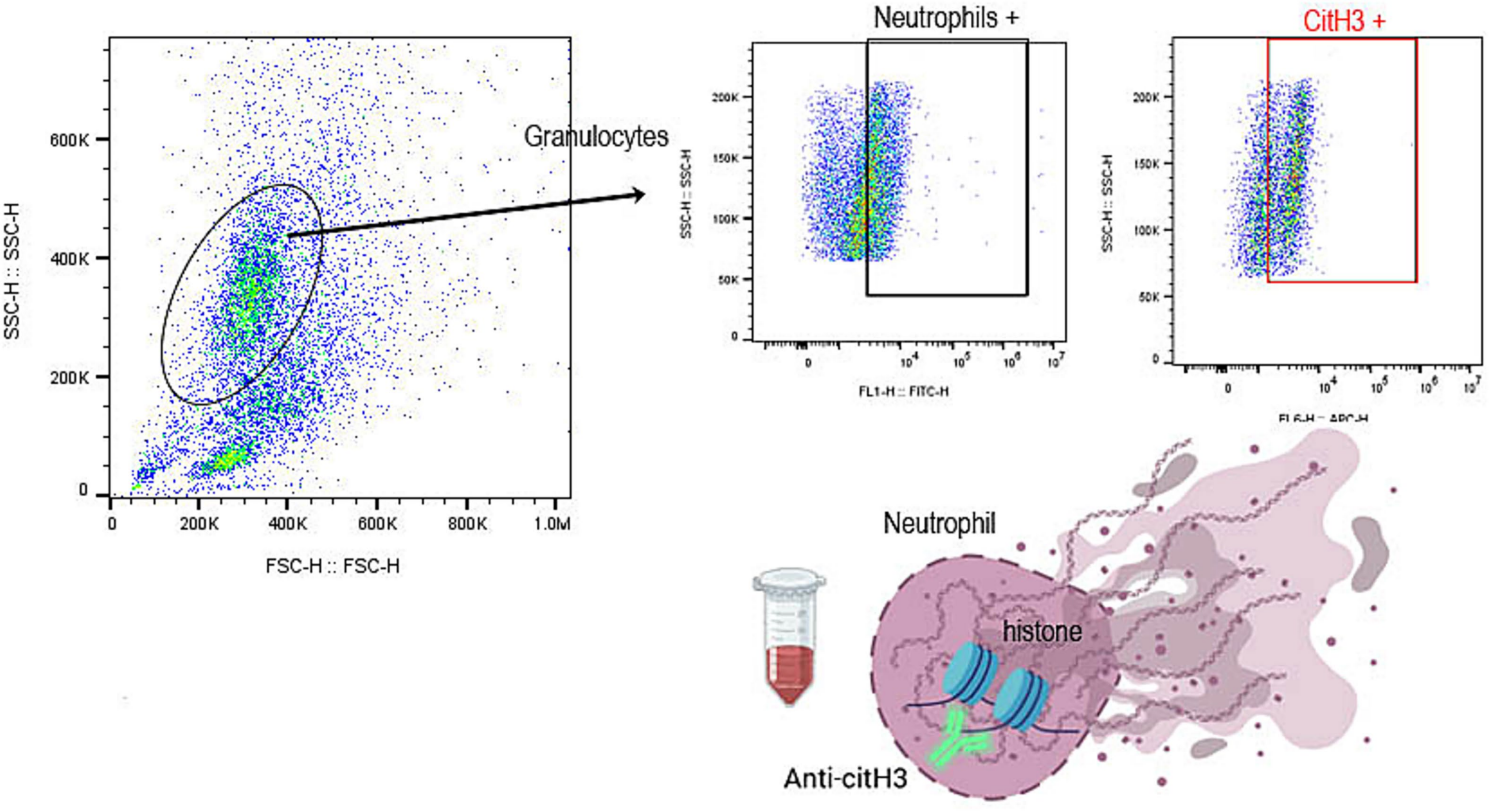
Representative scattered plots of diluted whole blood on flow cytometry. Neutrophils were standardized to 1 × 10^6^/mL, permeabilized, and washed before immunolabeling of neutrophils and citrullinated histones H3. Neutrophils were identified within the granulocyte gate (oval) and labeled positive for fluorescein isothiocynate-conjugated anti-dog neutrophil antibody. Citrullinated histone H3 positive neutrophils (red square) were quantified and measured as % positive neutrophils.

All flow cytometry analyses were conducted on a 3-laser flow cytometer (Cytoflex, Beckman Coulter, Brea, CA). Compensation for spectral overlaps was achieved using paired positive and negative sets of compensation beads (BD Life Sciences, Milpitas, CA) with mouse immunoglobulin G1 kappa isotype controls conjugated to the fluorophores used in this study (Invitrogen, Waltham, MA) under identical experimental conditions. Compensation matrices were applied using a commercially available Flowjo^™^ v10.10.0 Software (BD Life Sciences, Milpitas, CA). Gating strategies were based on previously established protocols (25) by utilizing fluorescence-minus-one (FMO) controls consisting of single immunolabeling of CD61, CD18, or neutrophils. Gating for leukocytes and granulocytes was determined by previously established forward- and side- scatter properties as described (Figure 2) (25). Leukocytes were identified as CD18 positive events out of a total of 100,000 events within the leukocyte gate (Figure 2A) and neutrophils were identified within the granulocyte gate (Figure 2B) as immunopositive cells for the anti-dog neutrophil marker. PLA and PNA were identified as double positive events consisting of CD18 or neutrophil positive cells and CD61 within the respective leukocyte and neutrophil gates (Figures 2A,B). Citrullinated histone H3 positive neutrophils were identified in permeabilized neutrophils gate (Figure 3). Flow cytometry analysis was performed using commercially available software (Flowjo^™^ v10.10.0, BD Life Sciences, Milpitas, CA).

### 2.7 Statistical analysis

Based on preliminary data on PNA in dogs on flow cytometry, an anticipated within subject 50% decrease due to heparin therapy would require 10 dogs to have an 80% power and an alpha of 0.05. With the anticipation that 10% of samples would undergo *in vitro* activation, 11 healthy student or staff-owned dogs were enrolled. Normality of data was assessed visually and by Shapiro–Wilk normality test. Dose-dependent effect for either NACH or UFH was evaluated using repeated measures ANOVA or Friedman test, followed by post-hoc multiple comparisons using Tukey’s test. Pair-wise comparisons were conducted using paired *t*-test or Wilcox signed rank test as appropriate. A *p*-value 0.05 was considered statistically significant. Data were analyzed using commercially available software (Prism 10.0, GraphPad Software, La Jolla, CA).

## 3 Results

### 3.1 Population demographic

A total of 11 dogs were studied. The median age of dogs was 8 years (range 2.0–12.0). Of the 11 dogs, four were females spayed and seven were male neutered. Two dogs were mixed-breeds and nine were pure-bred including one Chihuahua, one Great Dane, one Rottweiler, one Catahoula Leopard, two Pitbull Terriers and three Labrador Retrievers.

### 3.2 Complete blood counts

#### 3.2.1 High-dose NACH and UFH restored neutrophil counts, despite decreases in platelet count induced by *E. coli* and calf-thymus histones

Table 1 summarizes the selected hematological variables. Total leukocyte and lymphocyte count did not change significantly with any treatment. Neutrophil counts significantly decreased in the presence of heat-killed *E. coli* in vehicle controls and NACH 1 μg/mL when compared to unstimulated or resting control (*p* < 0.05). Compared to baseline measurements, platelet counts were significantly decreased in the absence of stimulants like *E. coli* and calf-thymus histones, indicating a decrease in platelet count over the incubation period. Treatment with *E. coli* and histones resulted in a significant decrease in measured platelet counts, consistent with agonist-induced platelet activation and aggregation. While UFH had variable effects, NACH at 5 μg/mL significantly attenuated this decrease in both inflammatory models, suggesting a protective effect against platelet consumption/aggregation. All concentrations of UFH did not change platelet count in the presence of *E. coli* while UFH at 0.4 and 40 resulted in insignificant decreases in the presence of histones.

**Table 1:** Summary of selected hematological variables in canine whole blood stimulated with E.coli or calf-thymus histones and treated in the presence or absence of non-anticoagulated heparin (NACH) and unfractionated heparin (UFH).

				NACH (μg/mL)				UFH (IU/mL)			
Hematological variables	Units	Baseline	Resting	0	1	5	10	0	0.4	4	40
*E. coli*											
White blood cell count (RI: 5.32–16.50)	×10^3^/μL	6.27 (6.01–8.52)	6.38 (5.28–8.17)	5.8 (5.43–7.58)	6.09 (4.75–7.94)	6.38 (5.20–7.45)	6.1 (4.91–7.32)	5.88 (5.43–7.58)	6.04 (5.07–7.50)	6.26 (4.84–7.18)	5.9 (5.16–8.28)
White blood cell count (RI: 3.00–14.30)	×10^3^/μL	4.21 (3.42–5.38)	4.34 (3.38–5.92)	3.91^b^ (3.08–5.32)	3.78^c^ (2.74–5.68)	4.27 (2.87–6.11)	3.89 (2.70–5.80)	3.91^b^ (3.08–5.32)	3.82 (3.00–5.55)	4.21 (3.12–5.30)	3.95 (3.18–5.84)
Lymphocyte (RI: 0.70–4.95)	×10^3^/μL	1.81 (1.14–2.42)	1.3 (0.89–2.42)	1.28 (0.87–2.30)	1.39 (1.01–2.15)	1.34 (0.95–2.15)	1.31 (1.00–2.15)	1.28 (0.87–2.30)	1.31 (0.95–2.27)	1.34 (0.95–2.28)	1.22 (0.98–2.31)
Platelet (RI: 100–500)	×10^3^/μL	191 (156–206)	151^a^ (130–163)	132^b^ (109–155)	131^c^ (98–149)	132 (105–147)	121^d^ (109–140)	132^b^ (109–155)	125^e^ (97–139)	125^f^ (106–150)	136^g^ (103–161)
Histone											
White blood cell (RI: 5.32–16.50)	×10^3^/μL	6.27 (6.01–8.52)	6.38 (5.28–8.17)	6.41 (5.65–7.48)	6.34 (5.42–7.42)	6.31 (5.25–7.92)	6.19 (5.33–8.04)	6.41 (5.65–7.48)	6.12 (5.14–7.77)	6.45 (5.12–7.63)	6.22 (5.36–7.92)
Neutrophil (RI: 3.00–14.30)	×10^3^/μL	4.21 (3.42–5.38)	4.34 (3.38–5.92)	4.26 (3.16–5.69)	4.06 (3.15–6.38)	4.14 (2.97–6.17)	4.25 (3.18–5.87)	4.26 (3.16–5.69)	4.04 (3.19–5.95)	4.26 (3.02–5.64)	4.09 (3.04–6.11)
Lymphocyte (RI: 0.70–4.95)	×10^3^/μL	1.81 (1.14–2.42)	1.3 (0.89–2.42)	1.42 (0.92–2.25)	1.38 (1.10–2.36)	1.39 (0.96–2.33)	1.32 (0.99–2.23)	1.42 (0.92–2.25)	1.3 (1.0–2.30)	1.41 (0.85–2.16)	1.43 (1.03–2.21)
Platelet count (RI: 100–500)	×10^3^/μL	191 (156–206)	151^a^ (130–163)	132^b^ (101–156)	132^c^ (109–155)	144 (108–163)	132^e^ (107–150)	132^b^ (101–156)	134 (107–165)	130^f^ (106–169)	135 (99–169)

RI = reference interval. *p* < 0.05. All data present as medians (25th to 75th percentile).

a Baseline vs. resting.

b Resting vs. vehicle control.

c Resting vs. 1 μg/mL NACH.

d Resting vs. 10 μg/mL NACH.

e Resting vs. 0.4 U/mL UFH.

f Resting vs. 4 U/mL UFH.

g Resting vs. 40 U/mL UFH.

### 3.3 *In vitro* Gram-negative (heated killed *E. coli*) sepsis model

#### 3.3.1 High-dose unfractionated heparin but not non-anticoagulant heparin modulated platelet–leukocyte aggregate formation

**Figure 4:**
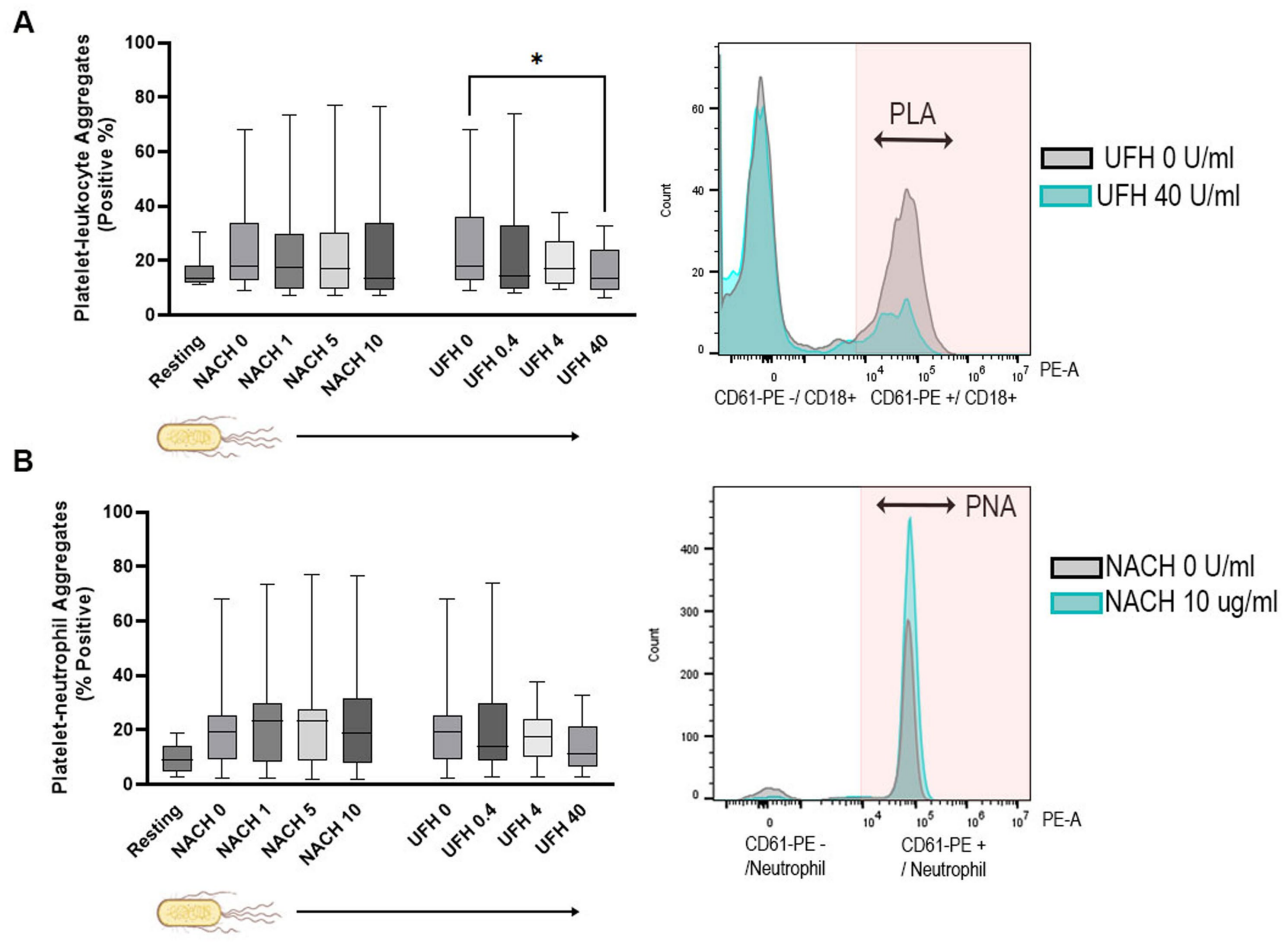
Box-and-whisker plots and representative histograms of platelet–leukocyte (PLA) and platelet–neutrophil aggregates (PNA) in canine whole blood treated with heat-killed *E. coli*. **(A)** No dose-dependent modulatory effects were noted with treatments of increasing concentrations of non-anticoagulated heparin (NACH 0, 1, 5, and 10 μg/mL) or unfractionated heparin (UFH 0, 0.4, 4, and 40 U/mL) in the presence of *E. coli*. However, the histogram demonstrates that high-dose UFH (40 U/mL) significantly decreases the overall number of PLA formation when compared to vehicle control. PLA are indicated by CD61-PE and CD18 positive cells in the pink shaded area. **(B)** No significant dose-dependent effects on PNA were found with either NACH or UFH with *E. coli* treatment. PLA are indicated by CD61-PE and neutrophil positive cells in the pink shaded area. Boxes represent the 25th and 75th percentile. Whiskers represent maximum and minimum, and lines within boxes represent medians. ^*^p < 0.05.

Dose-dependent effects of NACH and UFH on PLA formation were not noted (*p* = 0.42, *p* = 0.074, respectively). However, UFH at 40 U/mL was effective at decreasing PLA (13.65%, IQR: 9.0 to 24.05) compared to the vehicle control (UFH 0 U/mL = 18.10%, IQR: 12.68 to 36.18, *p* = 0.047) (Figure 4A). NACH and UFH did not have any dose-dependent effects on PNA formation (*p* = 0.26, *p* = 0.062, respectively) in the presence of *E. coli* (Figure 4B).

#### 3.3.2 Non-anticoagulant and unfractionated heparin modulated *E. coli*-induced neutrophil histone citrullination in a dose-dependent manner

**Figure 5:**
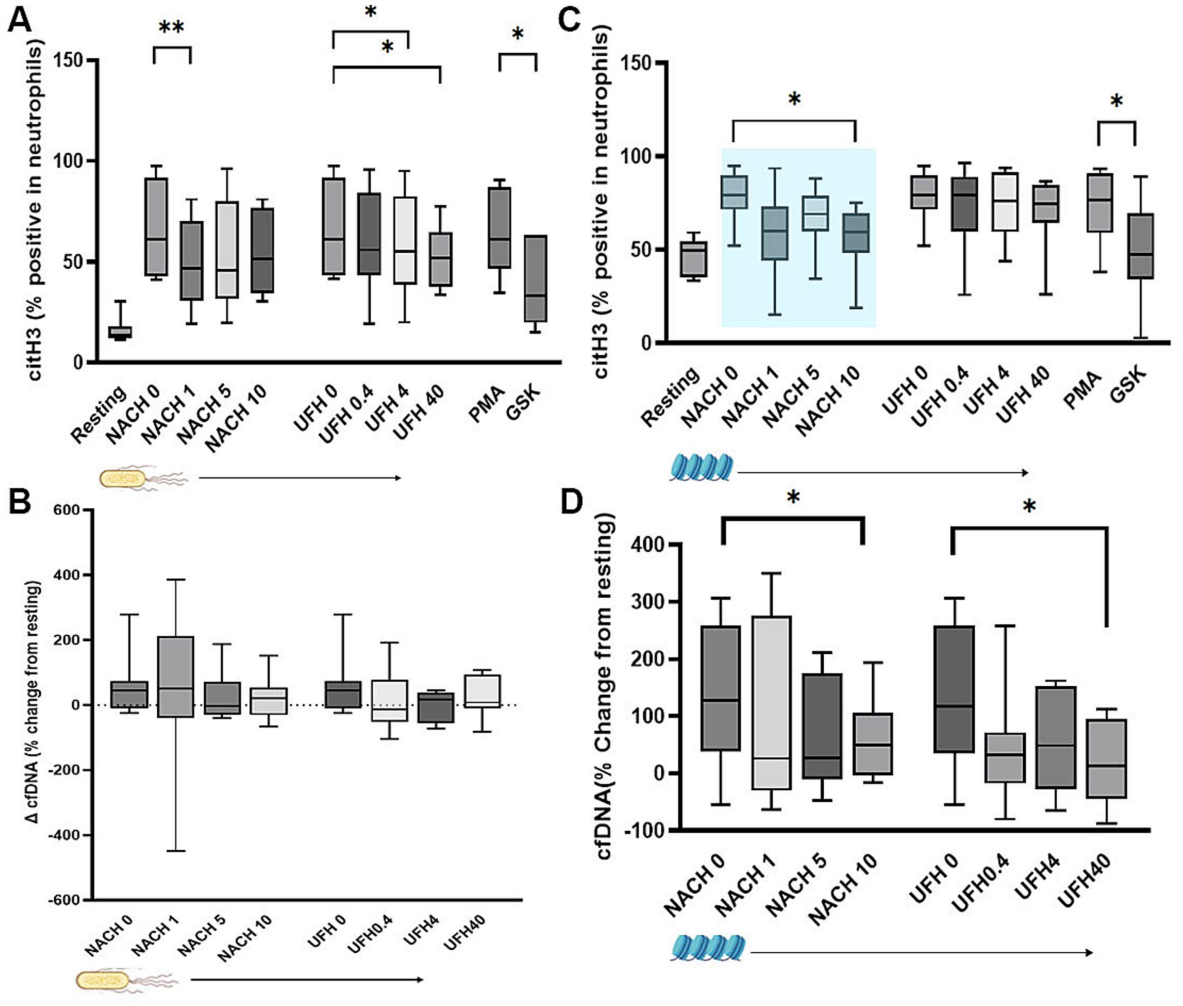
Neutrophil extracellular traps measured as intracellular citrullinated histone H3 (citH3) within neutrophils and cell-free DNA (cfDNA) in supernatant. **(A)** Box-and-whisker plots of citH3 expression within neutrophils (% positive) in recalcified diluted whole blood stimulated with heat-killed *E. coli* prior to treatments with increasing concentrations of non-anticoagulant heparin (NACH, 0, 1, 5 and 10 μg/mL) or unfractionated heparin (UFH 0, 4, 4 and 40 U/mL). Modulatory dose-dependent effects of NACH and UFH on citH3 expression were found with NACH at 1 μg/mL or UFH at 4 and 40 U/mL significantly decreased citH3 expression in neutrophils. PMA and GSK served as positive and negative controls, respectively. **(B)** cfDNA, measured by spectrophotometry and calculated based on changes from unstimulated or resting cells, did not change significantly despite treatments with NACH and UFH. **(C)** When whole blood was exposed to exogenous histones, only NACH demonstrated modulatory effects on citH3 expression. Additionally, NACH and UFH at their highest concentrations (10 μg/mL and 40 U/mL) were able to decrease cfDNA release in the presence of histones. Boxes represent the 25th and 75th percentile. Whiskers represent maximum and minimum and lines within boxes represent medians. Shaded box (pale blue) indicates a significant dose-dependent effect. ^*^p < 0.05.

To determine the dose-dependent effects of NACH and UFH on NET formation, neutrophil citrullination was evaluated by flow cytometry. Dose-dependent effects were noted with both NACH and UFH (*p* = 0.018, *p* = 0.027, respectively) (Figure 5A). NACH at 1 μg/mL significantly decreased intracellular citH3 (46.9%, IQR: 30.70 to 70.40) in comparison to the vehicle control (60.95%%, IQR: 43.40 to 91.50, *p* = 0.0058). Similarly, UFH at 4 U/mL and 40 U/mL was effective at decreasing intracellular citH3 (UFH 4 U/mL = 55%, IQR: 38.58 to 82.60 and UFH 40 U/mL = 51.85%, IQR: 37.55 to 64.43) in comparison to the vehicle control (*p* = 0.036, *p* = 0.020, respectively) (Figure 5A). Changes in cfDNA release did not differ significantly from unstimulated or resting cells in the presence of NACH (*p* = 0.72) or UFH (*p* = 0.60) (Figure 5B).

### 3.4 *In vitro* aseptic inflammation (calf-thymus histones) model

#### 3.4.1 High-dose non-anticoagulant heparin decreased platelet–neutrophil aggregate formation in a dose-dependent manner

**Figure 6:**
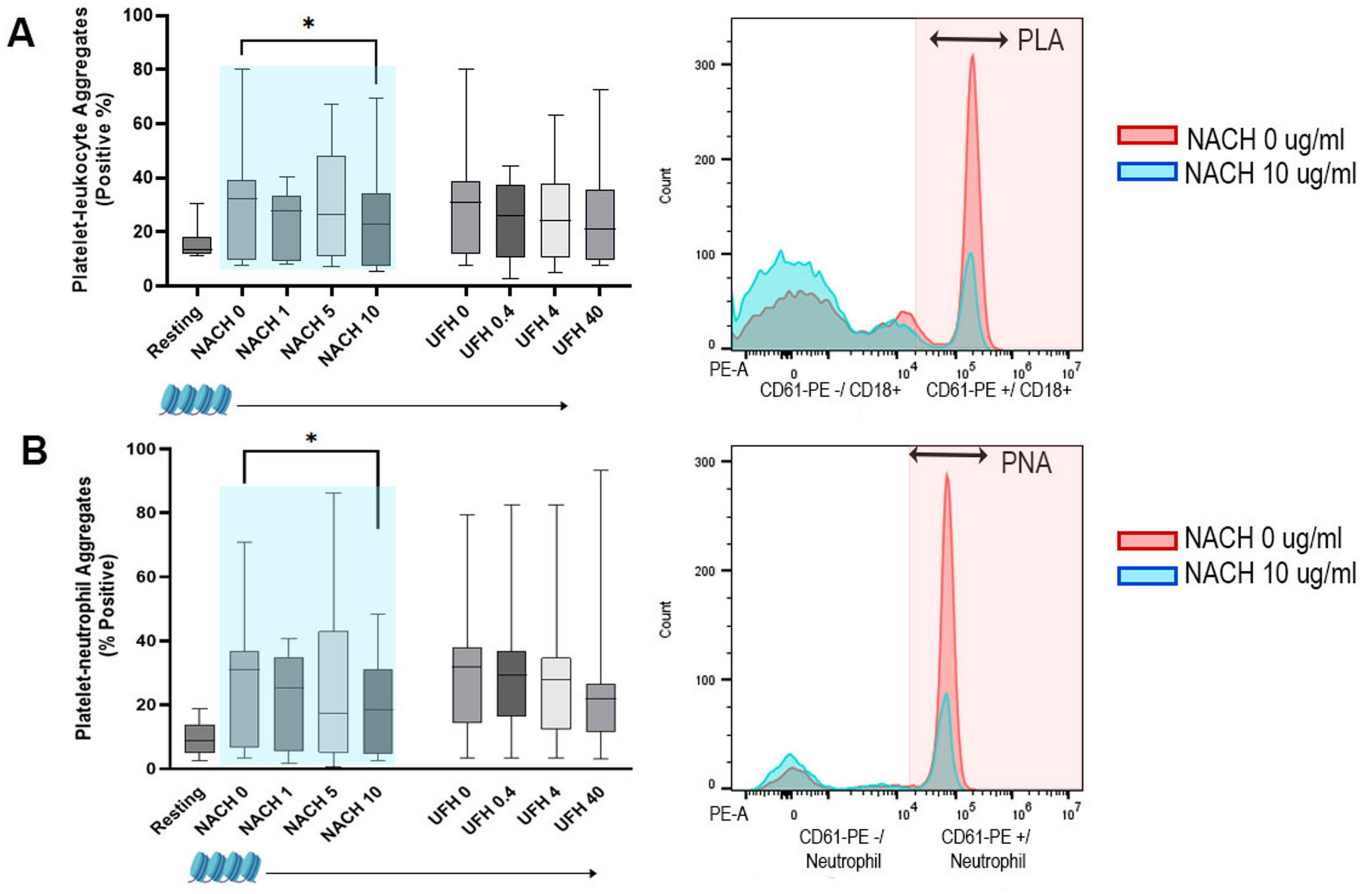
Box-and-whisker plots and representative histograms demonstrating the effects of non-anticoagulant heparin (NACH) on platelet–leukocyte (PLA) and platelet–neutrophil aggregates (PNA) formations in recalcified diluted canine whole blood treated with calf-thymus histones. Dose-dependent modulatory effects on PLA **(A)** and PNA **(B)** were found with NACH treatment in the presence of calf-thymus histones. NACH at 10 μg/mL was effective at decreasing formations of PLA **(A)** and PNA **(B)** as when compared to vehicle controls. Numbers of PLA and PNA are indicated in the respective histograms within the pink shaded areas. Boxes represent the 25th and 75th percentile. Whiskers represent maximum and minimum and lines within boxes represent medians. Shaded box (pale blue) indicates a significant dose-dependent effect. ^*^p < 0.05.

Whole blood treated with calf-thymus histones resulted in a significant increase in PLA (32.1%, IQR: 9.5 to 39.2) and PNA (31.05%, IQR: 6.75 to 36.73) compared to unstimulated (resting) samples (PLA = 13.35%, IQR: 12.05 to 18.08; PNA = 8.92%, IQR: 4.85 to 13.90, *p* = 0.015, respectively). While no dose-dependent effect of NACH on PLA formation was observed (*p* = 0.19), NACH at 10 μg/mL significantly reduced PLA formation (23.0%, IQR: 7.2 to 34.4, *p* = 0.04) when compared to vehicle control (32.1%, IQR: 9.5 to 39.2) (Figure 6A). A dose-dependent decrease in PNA formation by NACH was observed (*p* = 0.029) with NACH at 10 μg/mL being the most effective at reducing PNA (18.55%, IQR: 4.57 to 31.03) when compared to the vehicle control (*p* = 0.020) (Figure 6B). Treatment of diluted blood with UFH had no effects on PLA and PNA formations in the presence of calf-thymus histones (*p* = 0.22, 0.059, respectively).

#### 3.4.2 High-dose non-anticoagulant heparin but not unfractionated heparin modulated neutrophil extracellular trap formation by decreasing neutrophil histone citrullination and cell-free DNA release

A dose-dependent effect of NACH on histone citrullination in neutrophils was found (*p* = 0.024) (Figure 5C). Specifically, NACH at 10 μg/mL (59.6%, IQR: 48.1 to 69.70) was effective at lowering neutrophil citH3 expression when compared to the vehicle control (79.2%, IQR: 71.40 to 90.10, *p* = 0.019). However, there was not a dose-dependent effect of UFH (*p* = 0.60) on neutrophil citH3 expression. Although we did not observe a dose-dependent effect on cfDNA release with either NACH (*p* = 0.16) or UFH (*p* = 0.38) treatment, NACH at 10 μg/mL (61.22% ± 71.30) and UFH at 40 U/mL (17.65% ± 71.54) significantly modulated cfDNA release compared to the vehicle control (132.6% ± 126) (*p* = 0.043, 0.048, respectively) (Figure 5D).

## 4 Discussion

We demonstrated that NACH was most consistent at inhibiting PLA and PNA formation in our *in vitro* sterile inflammation model mediated by histones. Furthermore, we found a dose-dependent inhibitory effect of NACH on NET formation by decreasing histone citrullination and cfDNA release in the presence of histones. However, compared to UFH, NACH was mostly ineffective at mitigating the interactions between platelets and leukocytes in our Gram-negative sepsis model. Together, these findings shed light onto the stimulus-dependent heparin effects and underscore the complexity of immunothrombotic mechanisms in sepsis and SIRS and suggest that not all inflammatory triggers respond equivalently to a single therapeutic strategy.

NACH and UFH are highly negatively charged glycosaminoglycans that have been shown to modulate inflammation due to their ability to selectively bind to multiple proteins, including selectins, chemokines and complement proteins. The broad binding capacity of heparins, largely due to their negatively charged structure, may also be linked to their sulfated domains, enabling them to bind to selectins at the cellular surface, as well as to other cell membrane proteins (27). Selectins, such as P-selectin, on activated platelets may play a role in mediating platelet–leukocyte or platelet–neutrophil interactions in dogs by binding to P-selectin glycoprotein ligand 1 on leukocytes. Additionally, heparins also bind to the negatively charged regions of glycoprotein 1b alpha (GP1ba), a key receptor on platelets that bind to von Willebrand factors and the neutrophil integrin, macrophage antigen 1 (MAC-1), potentially inhibiting the firm adhesive interactions between platelets and neutrophils. Because P-selectins and GP1ba on platelets play a key role in mediating interactions between leukocytes, particularly with neutrophils and platelets, the effects of UFH and NACH on PLA and PNA were evaluated. As expected, the selected doses of partially desulfated NACH were more effective and consistent than UFH at inhibiting PNA formation in the presence of extracellular histones. One plausible explanation for this finding is that high concentrations of sulfated NACH of up to 10 μg/mL may possess significantly greater histone-binding capacity compared to the UFH concentrations used in this study, which ranged from therapeutic to supratherapeutic concentrations. Histones can directly activate human platelets via Toll-like receptors (TLR) 2 and 4, augmenting plasma thrombin generation through platelet activation (28). Canine platelets also express functional TLR4 enabling them to sense not only endotoxins, but also DAMPs, such as high mobility group box-1, to prime and augment their response to physiologic agonists like adenosine diphosphate (29). Enhanced platelet activation leads to inside-out signaling that not only upregulates P-selectin expression but also induces conformational changes that increase the binding affinity of integrins and glycoproteins, both of which are crucial for PNA and PLA formations (30). Although platelet activation was not directly assessed in this study, the observed decreases in PLA and PNA were also reflected in the less pronounced reduction in platelet counts at higher concentrations of NACH and UFH. These findings suggest that NACH and, to a lesser extent, UFH, may modulate platelet activation either by binding directly to histones or by mitigating the effects of a proinflammatory microenvironment such as through reactive oxygen species scavenging or downregulating cytokines, selectins, TNF-α, as reported by Cassinelly and Naggi (31). Nonetheless, further studies are needed to fully elucidate these mechanisms.

While NETs play a crucial role in protecting the host by eliminating pathogens and limiting their ability to disseminate systemically, multiple studies have shown that excessive NET formation is detrimental due to their bystander damaging effects to tissues, intravascular thrombosis and multiple organ failure (32). Although neutrophils possess all the molecular tools to form NETs on their own, multiple sepsis models showed that *in vivo* NETosis is highly dependent on platelet–neutrophil interactions (33–35). Given the inhibitory effects of heparins on PNA formation, we assessed the effects of NACH and UFH on histone or *E. coli*-mediated NET formation. Peptidyl arginine deiminase 4 (PAD4), which is highly expressed in neutrophils, catalyzes the post-translational modification of histones through citrullination, leading to chromatin decondensation and the subsequent release of cell-free DNA (cfDNA) (26). Consistent with our findings on PNA formation, NACH also modulated histone H3 citrullination in the presence of extracellular histones. This further supports the notion that high-dose NACH may scavenge free histones and thereby mitigate platelet activation and platelet–neutrophil interactions—both essential steps for histone modification and NET formation. In contrast, clinically achievable concentrations of UFH did not elicit this effect. These findings have direct clinical relevance because heparins are known to interact strongly with histones to neutralize their cytotoxic and proinflammatory effects on immune cells and platelets (17). Wildhagen et al. (21) reported that heparin inhibits the cytotoxic activity of purified histones in a dose-dependent manner by forming heparin–histone complexes that lack cytotoxic properties. This interaction occurs through high-affinity electrostatic binding (10). Lower concentrations of heparin have been shown to be less effective at attenuating histone-induced inflammation, a pattern also observed in our study. Because histone concentrations have been associated with survival in *in vivo* models and clinical canine studies (15, 36, 37), our findings emphasize the importance of histone modulation and support the need for future studies addressing therapeutic strategies that target extracellular histones.

In this study, a dose-dependent modulatory effect on neutrophil histone citrullination was observed with both NACH and UFH in the presence of heat-killed *E. coli*. Because no modulatory effects of heparins on PNA were detected, histone citrullination induced by *E. coli* may occur independently of direct platelet–neutrophil interactions. While *E. coli* lipopolysaccharide is considered a relatively weak agonist for NETosis in canine neutrophils, *E. coli*, once phagocytized by neutrophils, induces a robust oxidative burst. Hydrogen peroxide, together with hypochlorite, generated by myeloperoxidase, is essential for PAD4 activation and subsequent NET formation (26, 38–40). Additionally, other mediators released from leukocytes and platelets in the presence of *E. coli* such as IL-8, HMGB1 and platelet activating factors, may further contribute to PAD4 activation and histone citrullination (41). Beyond their binding properties, heparins have been shown to inhibit ROS generation in granulocytes, which may be an additional mechanism by which NACH and UFH reduce histone citrullination in neutrophils (42).

High-dose NACH and UFH reduced cfDNA release in the presence of histones, the observed effects were variable. The more specific marker of NETosis, citH3, showed a clearer dose-dependent inhibition by NACH, supporting its role in modulating this pathway The inconsistency in cfDNA may be attributable to substantial inter-individual variability among canine subjects, as well as potential contamination with genomic DNA from *in vitro* cell shearing during centrifugation, apoptosis, or necrosis. The latter likely explains the absence of significant dose-dependent reductions in cfDNA in *E. coli*-treated blood. Measurement of cell-free nucleosomes or further characterization of DNA fragment size using capillary electrophoresis could help distinguish the source of cfDNA in these experiments.

Although NACH and UFH demonstrated significant effects in histone-mediated inflammation, similar effects were not observed in heat-killed *E. coli*-mediated inflammation. Only high dose UFH (40 U/mL) was able to modulate PLA formation under these conditions. These results differ from those reported by Huang et al. (16), who showed that heparin decreased inflammatory cytokines levels (TNF-α, IL-6 and IL-8) in a lipopolysaccharide-induced sepsis model. However, it is important to emphasize that in that study, whole blood was preconditioned first with NACH and UFH prior to the introduction of lipopolysaccharide, which may partly explain the discrepancy. Early exposure of blood to heparin may allow immediate attenuation of cellular receptor engagement with DAMPs and their downstream signaling. In addition, dose-dependent effects of *E. coli* were not evaluated prior to selecting the bacterial dose in the present study. This may have caused cellular necrosis or an overwhelming inflammatory stimulus, thereby limiting the ability of heparin to effectively engage their molecular targets and modulate platelet–leukocyte interactions.

Several limitations should be considered when interpreting the findings of this study. Firstly, only the highest concentrations of NACH and UFH consistently demonstrated significant modulatory effects across the two experimental models. At this point, it remains unclear whether the higher doses yield additional benefit or whether their inflammatory modulation reaches a plateau. In addition, the *in vitro* design may not fully capture the complexity of inflammatory and cellular interactions that occur *in vivo*, particularly in the absence of endothelial cells and physiological shear. The chronic effect of NACH on inflammation may not be captured in our model. Third, the absence of additional biomarkers such as interleukins or TNF-α, limits direct comparison with previous studies. Furthermore, the lack of a Gram-positive model may have reduced the translational potential of our findings since sepsis in dogs commonly involves both Gram-negative and Gram-positive organisms. Fourth, since we had to titrate our UFH concentrations to clinically unattainable concentrations, parallel coagulation assays were not performed and the effects of NACH on coagulation were not evaluated. While this is a limitation, our study provides preliminary data for future pharmacokinetics and *in vivo* studies to determine whether therapeutically effective concentrations of NACH can be achieved without detrimental anticoagulant effects in clinical dogs. Finally, the lack of dose-ranging experiments for heat-killed *E. coli* and histones may have resulted in an inflammatory stimulus too robust to detect modulatory effects of heparins, thereby limiting interpretation of their anti-inflammatory potential under these conditions. Future studies should include live *E. coli* with various endotoxin virulence, as well as Gram positive bacteria to fully elucidate the anti-inflammatory effects of NACH. Lastly, differences in breed and sex might have influenced the efficacies of UFH and NACH. However, the sample size is too small for any meaningful subgroup analysis in this study, which is a limitation.

In conclusion, the results of the current study suggest that partially desulfated NACH is effective at attenuating histone-mediated PLA, PNA, and NET formation in a canine whole blood model. The findings further indicate that NACH may be more effective than UFH at reducing PLA and PNA formation under histone-mediated inflammatory conditions, although this advantage was not observed in Gram-negative–induced inflammation. Higher doses of NACH and evaluation in Gram-positive septic models may be necessary to fully characterize its modulatory effects and clinical relevance. Overall, this study represents an important first step toward the development of novel therapeutic strategies targeting immunothrombosis in critically ill veterinary patients with sepsis, and related inflammatory conditions.

## Data Availability

The original contributions presented in the study are included in the article/supplementary material, further inquiries can be directed to the corresponding author.

## References

[1] Cortellini S DeClue AE Giunti M Goggs R Hopper K Menard JM . Defining sepsis in small animals. J Vet Emerg Crit Care. (2024) 34:97–109. doi: 10.1111/vec.13359, 38351524

[2] Spillane AM Haraschak JL Gephard SE Nerderman BE Fick ME Reinhart JM. Evaluating the clinical utility of the systemic inflammatory response syndrome criteria in dogs and cats presenting to an emergency department. J Vet Emerg Crit Care. (2023) 33:315–26. doi: 10.1111/vec.13293, 37087544

[3] Ciuffoli E Troìa R Bulgarelli C Pontiero A Buzzurra F Giunti M. New-onset organ dysfunction as a screening tool for the identification of sepsis and outcome prediction in dogs with systemic inflammation. Front Vet Sci. (2024) 11:1369533. doi: 10.3389/fvets.2024.1369533, 38638640 PMC11024354

[4] Evans L Rhodes A Alhazzani W Antonelli M Coopersmith CM French C . Surviving sepsis campaign: international guidelines for management of sepsis and septic shock 2021. Intensive Care Med. (2021) 47:1181–247. doi: 10.1007/s00134-021-06506-y, 34599691 PMC8486643

[5] Li RHL Tablin F. A comparative review of neutrophil extracellular traps in sepsis. Front Vet Sci. (2018) 5:291. doi: 10.3389/fvets.2018.00291, 30547040 PMC6280561

[6] Brinkmann V Reichard U Goosmann C Fauler B Uhlemann Y Weiss DS . Neutrophil extracellular traps kill bacteria. Science. (2004) 303:1532–5. doi: 10.1126/science.1092385, 15001782

[7] Jeffery U LeVine DN. Canine neutrophil extracellular traps enhance clot formation and delay lysis. Vet Pathol. (2018) 55:116–23. doi: 10.1177/0300985817699860, 28346125

[8] Martinod K Wagner DD. Thrombosis: tangled up in NETs. Blood. (2014) 123:2768–76. doi: 10.1182/blood-2013-10-463646, 24366358 PMC4007606

[9] Dewitte A Lepreux S Villeneuve J Rigothier C Combe C Ouattara A . Blood platelets and sepsis pathophysiology: a new therapeutic prospect in critical ill patients? Ann Intensive Care. (2017) 7:115. doi: 10.1186/s13613-017-0337-7, 29192366 PMC5709271

[10] Iba T Levi M Levy JH. Intracellular communication and immunothrombosis in sepsis. J Thromb Haemost. (2022) 20:2475–84. doi: 10.1111/jth.15852, 35979601 PMC9804233

[11] Maneta E Aivalioti E Tual-Chalot S Emini Veseli B Gatsiou A Stamatelopoulos K . Endothelial dysfunction and immunothrombosis in sepsis. Front Immunol. (2023) 14:1144229. doi: 10.3389/fimmu.2023.1144229, 37081895 PMC10110956

[12] Chen Z Zhang H Qu M Nan K Cao H Cata JP . Review: the emerging role of neutrophil extracellular traps in sepsis and sepsis-associated thrombosis. Front Cell Infect Microbiol. (2021) 11:653228. doi: 10.3389/fcimb.2021.653228, 33816356 PMC8010653

[13] Mauro KD Lambert MP Kowalska MA Thawley VJ Poncz M Otto CM. Dose escalation trial of desulfated heparin (ODSH) in septic peritonitis. Front Vet Sci. (2022) 9:862308. doi: 10.3389/fvets.2022.862308, 35498738 PMC9043859

[14] Swann JW Skelly BJ. Systematic review of prognostic factors for mortality in dogs with immune-mediated hemolytic anemia. J Vet Intern Med. (2015) 29:7–13. doi: 10.1111/jvim.12514, 25586014 PMC4858088

[15] Hogwood J Pitchford S Mulloy B Page C Gray E. Heparin and non-anticoagulant heparin attenuate histone-induced inflammatory responses in whole blood. PLoS One. (2020) 15:e0233644. doi: 10.1371/journal.pone.0233644, 32469940 PMC7259574

[16] Huang X Han S Liu X Wang T Xu H Xia B . Both UFH and NAH alleviate shedding of endothelial glycocalyx and coagulopathy in LPS-induced sepsis. Exp Ther Med. (2019) 19:913–22. doi: 10.3892/etm.2019.8285, 32010252 PMC6966138

[17] Cao M Qiao M Sohail M Zhang X. Non-anticoagulant heparin derivatives for COVID-19 treatment. Int J Biol Macromol. (2023) 226:974–81. doi: 10.1016/j.ijbiomac.2022.12.090, 36528145 PMC9749384

[18] Riffo-Vasquez Y Somani A Man F Amison R Pitchford S Page CP. A non-anticoagulant fraction of heparin inhibits leukocyte diapedesis into the lung by an effect on platelets. Am J Respir Cell Mol Biol. (2016) 55:554–63. doi: 10.1165/rcmb.2015-0172OC, 27181499

[19] Buijsers B Yanginlar C Maciej-Hulme ML de Mast Q van der Vlag J. Beneficial non-anticoagulant mechanisms underlying heparin treatment of COVID-19 patients. EBioMedicine. (2020) 59:102969. doi: 10.1016/j.ebiom.2020.102969, 32853989 PMC7445140

[20] Wang P Chi L Zhang Z Zhao H Zhang F Linhardt RJ. Heparin: an old drug for new clinical applications. Carbohydr Polym. (2022) 295:119818. doi: 10.1016/j.carbpol.2022.119818, 35989029

[21] Wildhagen KCAA García de Frutos P Reutelingsperger CP Schrijver R Aresté C Ortega-Gómez A . Nonanticoagulant heparin prevents histone-mediated cytotoxicity in vitro and improves survival in sepsis. Blood. (2014) 123:1098–101. doi: 10.1182/blood-2013-07-514984, 24264231

[22] De Laforcade AM Freeman LM Shaw SP Brooks MB Rozanski EA Rush JE. Hemostatic changes in dogs with naturally occurring sepsis. J Vet Intern Med. (2003) 17:674–9. doi: 10.1111/j.1939-1676.2003.tb02499.x, 14529134

[23] Levi M van der Poll T. Coagulation and sepsis. Thromb Res. (2017) 149:38–44. doi: 10.1016/j.thromres.2016.11.007, 27886531

[24] Pan Q Zhang C Wu X Chen Y. Identification of a heparosan heptasaccharide as an effective anti-inflammatory agent by partial desulfation of low molecular weight heparin. Carbohydr Polym. (2020) 227:115312. doi: 10.1016/j.carbpol.2019.115312, 31590876

[25] Duler L Visser L Nguyen N Johnson LR Stern JA Li RHL. Platelet hyperresponsiveness and increased platelet–neutrophil aggregates in dogs with myxomatous mitral valve disease and pulmonary hypertension. J Vet Intern Med. (2024) 38:2052–63. doi: 10.1111/jvim.17067, 38773707 PMC11256165

[26] Li RHL Ng G Tablin F. Lipopolysaccharide-induced neutrophil extracellular trap formation in canine neutrophils is dependent on histone H3 citrullination by peptidylarginine deiminase. Vet Immunol Immunopathol. (2017) 193–194:29–37. doi: 10.1016/j.vetimm.2017.10.002, 29129225

[27] Mohamed S Coombe D. Heparin mimetics: their therapeutic potential. Pharmaceuticals. (2017) 10:78. doi: 10.3390/ph10040078, 28974047 PMC5748635

[28] Eustes AS Ahmed A Swamy J Patil G Jensen M Wilson KM . Extracellular histones: a unifying mechanism driving platelet-dependent extracellular vesicle release and thrombus formation in COVID-19. J Thromb Haemost. (2024) 22:2514–30. doi: 10.1016/j.jtha.2024.05.019, 38815756 PMC11343660

[29] Li RHL Nguyen N Tablin F. Canine platelets express functional Toll-like receptor-4: lipopolysaccharide-triggered platelet activation is dependent on adenosine diphosphate and thromboxane A2 in dogs. BMC Vet Res. (2019) 15:245. doi: 10.1186/s12917-019-1997-3, 31307465 PMC6632210

[30] Xu X Wang Y Tao Y Dang W Yang B Li Y. The role of platelets in sepsis: a review. Biomol Biomed. (2024) 24:741–52. doi: 10.17305/bb.2023.10135, 38236204 PMC11293227

[31] Cassinelli G Naggi A. Old and new applications of non-anticoagulant heparin. Int J Cardiol. (2016) 212:S14–21. doi: 10.1016/S0167-5273(16)12004-2, 27264866

[32] Mutua V Gershwin LJ. A review of neutrophil extracellular traps (NETs) in disease: potential anti-NETs therapeutics. Clin Rev Allergy Immunol. (2021) 61:194–211. doi: 10.1007/s12016-020-08804-7, 32740860 PMC7395212

[33] Carestia A Kaufman T Schattner M. Platelets: new bricks in the building of neutrophil extracellular traps. Front Immunol. (2016) 7:271. doi: 10.3389/fimmu.2016.00271, 27458459 PMC4933697

[34] Clark SR Ma AC Tavener SA McDonald B Goodarzi Z Kelly MM . Platelet TLR4 activates neutrophil extracellular traps to ensnare bacteria in septic blood. Nat Med. (2007) 13:463–9. doi: 10.1038/nm1565, 17384648

[35] McDonald B Davis RP Kim S-J Tse M Esmon CT Kolaczkowska E . Platelets and neutrophil extracellular traps collaborate to promote intravascular coagulation during sepsis in mice. Blood. (2017) 129:1357–67. doi: 10.1182/blood-2016-09-741298, 28073784 PMC5345735

[36] Lawson C Smith SA O’Brien M McMichael M. Neutrophil extracellular traps in plasma from dogs with immune-mediated hemolytic anemia. J Vet Intern Med. (2018) 32:128–34. doi: 10.1111/jvim.14881, 29214674 PMC5787156

[37] Letendre J-A Goggs R. Concentrations of plasma nucleosomes but not cell-free DNA are prognostic in dogs following trauma. Front Vet Sci. (2018) 5:180. doi: 10.3389/fvets.2018.00180, 30105230 PMC6077184

[38] Du Preez K Rautenbach Y Hooijberg EH Goddard A. Oxidative burst and phagocytic activity of phagocytes in canine parvoviral enteritis. J Vet Diagn Invest. (2021) 33:884–93. doi: 10.1177/10406387211025513, 34148453 PMC8366246

[39] Pleskova SN Erofeev AS Vaneev AN Gorelkin PV Bobyk SZ Kolmogorov VS . ROS production by a single neutrophil cell and neutrophil population upon bacterial stimulation. Biomedicine. (2023) 11:1361. doi: 10.3390/biomedicines11051361, 37239032 PMC10216488

[40] Rohrbach AS Slade DJ Thompson PR Mowen KA. Activation of PAD4 in NET formation. Front Immunol. (2012) 3:360. doi: 10.3389/fimmu.2012.00360, 23264775 PMC3525017

[41] Li RHL Hommel C Nguyen N. Lipopolysaccharide-activated canine platelets upregulate high mobility group box-1 via Toll-like receptor 4. Front Vet Sci. (2021) 8:674678. doi: 10.3389/fvets.2021.674678, 34235204 PMC8255672

[42] Dandona P Qutob T Hamouda W Bakri F Aljada A Kumbkarni Y. Heparin inhibits reactive oxygen species generation by polymorphonuclear and mononuclear leucocytes. Thromb Res. (1999) 96:437–43. doi: 10.1016/S0049-3848(99)00132-2, 10632466

